# Characterizing the antimicrobial resistance profile of *Escherichia coli* found in sport animals (fighting cocks, fighting bulls, and sport horses) and soils from their environment

**DOI:** 10.14202/vetworld.2022.2673-2680

**Published:** 2022-11-25

**Authors:** Tuempong Wongtawan, Ruethai Narinthorn, Narin Sontigun, Chalutwan Sansamur, Yotsapat Petcharat, Punpichaya Fungwithaya, Phirabhat Saengsawang, Patrick J. Blackall, Thotsapol Thomrongsuwannakij

**Affiliations:** 1 Akkhraratchakumari Veterinary College, Walailak University, Thai Buri, Tha Sala, Nakhon Si Thammarat 80160, Thailand; 2 Centre for One Health, Walailak University, Thai Buri, Tha Sala, Nakhon Si Thammarat 80160, Thailand; 3 Excellence Centre for Melioidosis and other microorganisms, Walailak University, Thai Buri, Tha Sala, Nakhon Si Thammarat 80160, Thailand; 4 Queensland Alliance for Agriculture and Food Innovation, The University of Queensland, St Lucia 4067, Australia

**Keywords:** antimicrobial resistance, beta-lactamase, bulls, *Escherichia coli*, trimethoprim

## Abstract

**Background and Aim:**

Antimicrobial resistance (AMR) is a significant threat to global health and development. Inappropriate antimicrobial drug use in animals cause AMR, and most studies focus on livestock because of the widespread use of antimicrobial medicines. There is a lack of studies on sports animals and AMR issues. This study aimed to characterize the AMR profile of *E. coli* found in sports animals (fighting cocks, fighting bulls, and sport horses) and soils from their environment.

**Materials and Methods:**

Bacterial isolation and identification were conducted to identify *E. coli* isolates recovered from fresh feces that were obtained from fighting cocks (n = 32), fighting bulls (n = 57), sport horses (n = 33), and soils from those farms (n = 32) at Nakhon Si Thammarat. Antimicrobial resistance was determined using 15 tested antimicrobial agents - ampicillin (AM), amoxicillin-clavulanic acid, cephalexin (CN), cefalotin (CF), cefoperazone, ceftiofur, cefquinome, gentamicin, neomycin, flumequine (UB), enrofloxacin, marbofloaxacin, polymyxin B, tetracycline (TE), and sulfamethoxazole/trimethoprim (SXT). The virulence genes, AMR genes, and phylogenetic groups were also examined. Five virulence genes, *iroN*, *ompT*, *hlyF*, *iss*, and *iutA*, are genes determining the phylogenetic groups, *chuA*, *cjaA*, and *tspE4C2*, were identified. The AMR genes selected for detection were *bla*TEM and *bla*SHV for the beta-lactamase group; *cml-A* for phenicol; *dhfrV* for trimethoprim; *sul1* and *sul2* for sulfonamides; *tetA, tetB*, and *tetC* for TEs; and *qnrA, qnrB*, and *qnrS* for quinolones.

**Results:**

The *E. coli* derived from sports animals were resistant at different levels to AM, CF, CN, UB, SXT, and TE. The AMR rate was overall higher in fighting cocks than in other animals, with significantly higher resistance to AM, CF, and TE. The highest AMR was found in fighting cocks, where 62.5% of their isolates were AM resistant. In addition, multidrug resistance was highest in fighting cocks (12.5%). One extended-spectrum beta-lactamase *E. coli* isolate was found in the soils, but none from animal feces. The phylogenetic analysis showed that most *E. coli* isolates were in Group B1. The *E. coli* isolates from fighting cocks had more virulence and AMR genes than other sources. The AMR genes found in 20% or more of the isolates were *bla*TEM (71.9%), *qnrB* (25%), *qnrS* (46.9%), and *tetA* (56.25%), whereas in the *E. coli* isolates collected from soils, the only resistance genes found in 20% or more of the isolates were *bla*TEM (30.8%), and *tetA* (23.1%).

**Conclusion:**

*Escherichia coli* from fighting cock feces had significantly higher resistance to AM, CF, and TE than isolates from other sporting animals. Hence, fighting cocks may be a reservoir of resistant *E. coli* that can transfer to the environment and other animals and humans in direct contact with the birds or the birds’ habitat. Programs for antimicrobial monitoring should also target sports animals and their environment.

## Introduction

Antimicrobial resistance (AMR) is one of the top ten global public health threats facing humanity [1]. Antimicrobial misuse and overuse are the main factors contributing to the developing drug-resistant pathogens [2, 3]. Antimicrobial resistance has a substantial economic burden on the economy since it may cause mortality and disability, and prolonged sickness necessitates longer hospital stays, more costly medicines, and financial pressure on the afflicted individual [4].

One aspect thought to have accelerated AMR is inappropriate antimicrobial drug use in animals, and the bulk of research in this area has been concentrated on livestock [5]. However, examining AMR in companion animals is also vital, as several antimicrobial drugs used in companion animals are also used in humans [6–8]. In addition, companion animals and humans frequently interact through different activities such as petting, training, playing, and feeding. These interactions may raise the risk of transmitting various pathogens that infect these animals, including parasites [9], bovine tuberculosis [10], dermatophytes [11], and highly pathogenic avian influenza [12], and AMR genes [13].

In Southern Thailand, fighting cocks, fighting bulls, and riding horses are considered valuable companions and sports animals. They have long been linked with Thai culture [14, 15] and are recently becoming employed in tourism. Not only are sports animals bred for entertainment, but they also contribute substantially to family income, mainly from fighting bulls and cocks [16]. Bullfighting in Thailand is distinct from bullfighting in other countries (e.g., Spain, Portugal, and France); it is a contest between bulls, not humans [17]. Thailand bullfighting is seldom fatal but often results in minor injuries (skin wounds) that may require antimicrobial treatment [17]. Cockfighting is popular not just in Thailand [18] but also in many other Asian nations, including India, Bangladesh, and Myanmar [18].

*Escherichia coli* is present in most animals, and a significant amount of research on AMR in *E. coli* has been conducted in livestock (e.g., broilers, dairy cows, and pigs), showing a high incidence of AMR across several drug classes [19, 20]. There are reports of resistance to many antimicrobial drugs against *E. coli* from companion animals and pets such as dogs, cats, and horses [21, 22]. Regarding sports animals, there are studies on AMR in bacteria from horses and most of these studies have occurred in Europe and North America [23, 24]. Few investigations on *E. coli* obtained from horses have been conducted in Asia, specifically in South Korea and Japan [25, 26]. There appear to have been no studies on fighting cocks or fighting bulls. Notably, the occurrence of extended-spectrum β-lactamases (ESBLs) has become common in *E. coli* recovered from animals and humans globally. This organism is usually multidrug-resistant, causing a difficult problem to treat and burdening the health systems and patient health [27–29]. Because ESBL-producing bacteria dynamically circulate across humans, animals, food, and the environment, these organisms have become a general concern from the viewpoint of One Health [27].

This study aimed to characterize the AMR profile of *E. coli* found in sports animals (fighting cocks, fighting bulls, and sport horses) and soils from their environment. Antibiotic-resistant *E. coli* from these sources may transfer from animals/environment to humans. There is currently no report on AMR in sports animals in Thailand, and this study will contribute to increasing awareness of AMR in both sports animals and people.

## Materials and Methods

### Ethical approval

The use of animals in this study was approved by the Institutional Animal Care and Use Committee of Walailak University (ID: 63023).

### Study period and location

This research was conducted for 12 months (January–December 2021), consisting of pre-research, experimental, and laboratory examination. The samples were collected from fighting cocks, fighting bulls, sport horses, and soils from the same farms, with all sites being located in Nakorn Si Thammarat, Thailand. The laboratory examinations were conducted at Walailak University.

### Sample collection

Fresh feces were obtained from fighting cocks (n = 32), fighting bulls (n = 57), sports horses (n = 33), and soils (n = 32) at Nakhon Si Thammarat, the region of southern Thailand that holds the main population of sport animals. The soils were obtained from the same environments as the fighting cocks (n = 9), the fighting bulls (n = 17), and the sport horses (n = 6).

### *Escherichia coli* isolation and identification

Plates of 5% sheep blood agar (SBA) (Oxoid, Hampshire, England) and MacConkey agar (Oxoid, Hampshire) were used to incubate samples for 24–48 h at 37°C. Pink colonies on MacConkey agar were subcultured onto eosin methylene blue (EMB) agar (Oxoid, Hampshire) and incubated aerobically for 24 h at 37°C. On EMB agar, presumptive colonies using metallic green sheen were subcultured onto SBA and incubated at 37°C for 24 h.

The suspect *E. coli* isolates were presumptively found using biochemical tests, including oxidase, indole, and triple sugar iron tests. The presumptive *E. coli* isolates were then stored at −80°C in tryptone soya broth (Oxoid, Hampshire) containing 20% glycerol for further study. Finally, *E. coli* was confirmed using the VITEK^®^ 2 card for Gram-negative Organisms in conjunction with the VITEK^®^ 2 COMPACT machine (bioMérieux, Marcy l’Etoile, France).

### Antimicrobial resistance profile

The AMR was evaluated using a minimum inhibitory concentration based automated system, specifically the VITEK^®^ 2 AST-GN96 test kit cards and VITEK^®^ 2 COMPACT machine (bioMérieux). The AMR was determined according to the Clinical and Laboratory Standards Institute guidelines [30]. The 15 antimicrobial agents tested were ampicillin (AM), amoxicillin-clavulanic acid (AMC), cephalexin (CN), cefalotin (CF), cefoperazone (CFP), ceftiofur (CFT), cefquinome (CEQ), gentamicin (GM), neomycin (N), flumequine (UB), enrofloxacin (ENR), marbofloxacin (MRB), polymyxin B (PB), tetracycline (TE), and sulfamethoxazole/trimethoprim (SXT).

### Detection of virulence genes, AMR genes, and phylogenetic group determination

Five virulence genes, *iroN*, *ompT*, *hlyF*, *iss*, and *iutA*, were discovered using a pentaplex polymerase chain reaction (PCR) as previously described [31]. Genes *chuA*, *cjaA*, and *tspE4C2* were used to determine the phylogenetic groups as previously described [32]. *Escherichia coli* was categorized into four major phylogenetic groups (A, B1, B2, and D).

The AMR genes selected for detection and the relevant published detection assays used were *bla*TEM and *bla*SHV for the beta-lactamase group; *cml-A* for phenicol; *dhfrV* for trimethoprim [33]; *sul1* and *sul2* for sulfonamides [34, 35]; *tetA, tetB*, and *tetC* for the TEs [36]; *qnrA, qnrB*, and *qnrS* for quinolones [37, 38].

Positive samples for each gene were confirmed by sequencing using the appropriate PCR primers, and these DNA preparations were then used as PCR-positive controls.

### Polymerase chain reaction and electrophoresis

DNA extraction was performed using a bacterial genomic DNA kit (Geneaid, New Taipei City, Taiwan), in accordance with the manufacturer’s instructions as previously described [39]. The PCR was performed using KAPA2G Fast HotStart ReadyMix (Roche, Basel, Switzerland) following the product’s instruction. The PCR machine was GeneAmp PCR System 9700 (Thermo Fisher Scientific, MA, USA). The PCR product was confirmed by electrophoresis (Major Science, CA, USA) using a 1.5% agarose gel. DNA was stained with FluoroVue™ (SMOBIO, Hsinchu City, Taiwan) and visualized using ultraviolet transilluminator G-BOX F3 Gel imaging machine (G-BOX F3, Syngene, Cambridge, UK). The primer sequence, amplicon size, and annealing temperature are indicated in Table-1 [32–38].

**Table-1:** Primers used for the amplification of phylogenetic grouping and antimicrobial resistance genes.

Group	Gene name	Primer sequence	Amplicon size (bp)	Annealing temperature (°C)	Reference
Phylogenetic group	*chuA*	F: 5’-GACGAACCAACGGTCAGGAT-3’	279	55	[32]
		R: 5’-TGCCGCCAGTACCAAAGACA-3’			
	*yjaA*	F: 5’-TGAAGTGTCAGGAGACGCTG-3’	211	55	[32]
		R: 5’-ATGGAGAATGCGTTCCTCAAC-3’			
	*tspE4C2*	F: 5’-GAGTAATGTCGGGGCATTCA-3’	152	55	[32]
		R: 5’-CGCGCCAACAAAGTATTACG-3’			
Antimicrobial groups					
Beta-lactams	*bla*TEM	F: 5’-GAGTATTCAACATTTTCGT-3’	857	58	[33]
		R: 5’-ACCAATGCTTAATCAGTGA-3’			
	*bla*SHV	F: 5’-TCGCCTGTGTATTATCTCCC-3’	768	58	[33]
		R: 5’-CGCAGATAAATCACCACAATG-3’			
Phenicols	*cml-A*	F: 5’-CCGCCACGGTGTTGTTGTTATC-3’	698	58	[33]
		R: 5’-CACCTTGCCTGCCCATCATTAG-3’			
Tetracyclines	*tetA*	F: 5’-GTAATTCTGAGCACTGTCGC-3’	956	57	[36]
		R: 5’-CTGCCTGGACAACATTGCTT-3’			
	*tetB*	F: 5’-CTCAGTATTCCAAGCCTTTG-3’	414	52	[36]
		R: 5’-ACTCCCCTGAGCTTGAGGGG-3’			
	*tetC*	F: 5’-CCTCTTGCGGGATATCGTCC-3’	505	65	[36]
		R: 5’-GGTTGAAGGCTCTCAAGGGC-3’			
Trimethoprim	*dhfrV*	F: 5’-AAGAATGGAGTTATCGGGAATG-3’	391	58	[33]
		R: 5’-GGGTAAAAACTGGCCTAAAATTG-3’			
Sulfonamides	*sul1*	F: 5’-CTTCGATGAGAGCCGGCGGC-3’	433	58	[35]
		R: 5’-GCAAGGCGGAAACCCGCGCC-3’			
	*sul2*	F: 5’-CGGCATCGTCAACATAACC-3’	720	58	[34]
		R: 5’-GTGTGCGGATGAAGTCAG-3’			
Quinolones	*qnrA*	F: 5’-TCAGCAAGAGGATTTCTCA-3’	657	48	[38]
		R: 5’-GGCAGCACTATTACTCCCA-3’			
	*qnrB*	F: 5’-GATCGTGAAAGCCAGAAAGG-3’	469	53	[37]
		R: 5’-ACGATGCCTGGTAGTTGTCC-3’			
	*qnrS*	F: 5’-ACGACATTCGTCAACTGCAA-3’	417	53	[37]
		R: 5’-TAAATTGGCACCCTGTAGGC-3’			

### Statistical analysis

Statistical analysis was conducted using R-programming language version 4.0.2 [40]. The Pearson’s Chi-square test was used to investigate the relationship between resistance rate and AMR genes among cocks, bulls, horses, and soils. In addition, the Pearson’s Chi-square test was also used to examine the relationship among the phylogenetic group of *E. coli* isolates from cocks, bulls, horses, and soils. The Bonferroni correction was used as a multiple comparison analysis to test the significant factors from univariate analysis. All statistical analyses were conducted under a 95% confidence interval, and p < 0.05 was considered a significant level.

## Results

### *Escherichia coli* isolates

A total of 32, 57, 33, and 26 *E. coli* isolates were obtained from fighting cocks, fighting bulls, sport horses, and soils, respectively.

### Phylogenetic group

The phylogenetic grouping of the isolates is indicated in Table-2. The largest phylogenetic group of the *E. coli* isolates was Group B1 for the fighting cocks, fighting bulls, sport horses, and soils (40.6%, 59.7%, 72.7%, and 42.3%, respectively). However, no significant difference (p > 0.05) in the phylogenetic groups among fighting cocks, fighting bulls, sport horses, and soils was found.

**Table-2:** Phylogenetic grouping of *E. coli* isolated from fighting cocks, fighting bulls, sport horses, and soils.

Phylogenetic group	*E. coli* isolates (%)			

	Fighting cocks (n=32)	Fighting bulls (n=57)	Sport horses (n=33)	Soils (n=26)
A	28.1	12.3	12.1	19.2
B1	40.6	59.7	72.7	42.3
B2	12.5	3.5	0	3.9
D	18.8	24.6	15.2	34.62

*E. coli=Escherichia coli*

### Virulence genes

As indicated in Table-3, the *iroN* gene was the most frequently detected gene in fighting cocks (28.1%), sport horses (6.1%), and soils (15.4%). Alternatively, the most frequently found gene in fighting bulls was *iss* (8.8%). Interestingly, *E. coli* isolates from fighting bulls and sport horses showed a lower prevalence of all five virulence genes present in the isolates from fighting cocks and soils. The only significant difference (p < 0.05) in the virulence genes was the prevalence of *hlyF* between isolates from fighting cocks and fighting bulls.

**Table-3:** Prevalence of virulence genes in *E. coli* isolated from fighting cocks, fighting bulls, sport horses, and soils.

Virulence genes	*E. coli* isolates harboring virulence genes (%)			

	Fighting cocks (n=32)	Fighting bulls (n=57)	Sport horses (n=33)	Soils (n=26)
*iroN*	28.1	7.0	6.1	15.4
*hlyF*	25.0	3.5	3.0	11.5
*iutA*	15.6	1.8	0.0	3.9
*ompT*	15.6	1.8	3.0	11.5
*iss*	15.6	8.8	3.0	11.5

*E. coli=Escherichia coli*

### Phenotypic AMR

**Figure-1:**
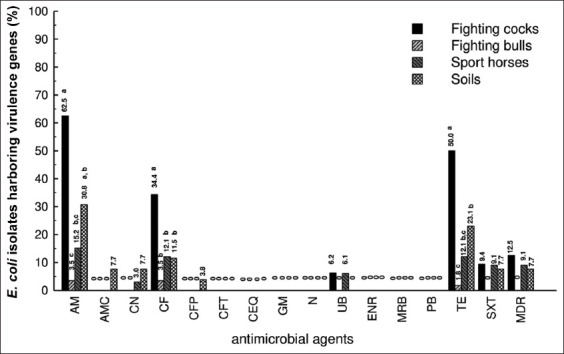
The frequency of resistance to fifteen antimicrobial agents in *Escherichia coli* isolated from fighting cocks (n = 32), fighting bulls (n = 57), sport horses (n = 33), and soils (n = 26). AM=Ampicillin, AMC=Amoxicillin/clavulanic acid, CN=Cefalexin, CF=Cefalotin, CFP=Cefoperazone, CFT=Ceftiofur, CEQ=Cefquinome, GM=Gentamicin, N=Neomycin, UB=Flumequine, ENR=Enrofloxacin, MRB=Marbofloxacin, PB=Polymyxin B, TE=Tetracycline, SXT=Trimethoprim/sulfamethoxazole, MDR=Multidrug resistance. Different superscripts in each column mean statistically significant difference (p < 0.05).

The AMR profile and the significant difference (p < 0.05) among the isolates from fighting cocks, fighting bulls, sport horses, and soils are indicated in Figure-1. Overall, *E. coli* isolates from fighting cocks had a significantly higher rate of AMR than AM (62.5%), CF (34.4%), and TE (50%) than isolates from the other sources. In addition, *E. coli* isolated from soils showed the second-highest resistance to AM (30.8%), CF (11.5%), and TE (23.1%). While most *E. coli* isolates from fighting bulls were susceptible to all antimicrobial agents, few resistant isolates with resistance to AM (3.5%), CF (3.5%), and TE (1.8%) were identified. None of the isolates were CFT-resistant to CFT, CEQ, GM, N, ENR, MRB, or PB.

Multidrug resistance isolates (i.e., bacteria resistant to three or more antimicrobial classes) were found in fighting cocks, sport horses, and soils (12.5%, 9.1%, and 7.7%, respectively) but were absent in the fighting bull isolates. Only one ESBL isolate was collected (from a soil sample), and this ESBL isolate was resistant to five drugs, AM, CN, CF, CFP, and TE.

### Antimicrobial resistance genes

**Figure-2:**
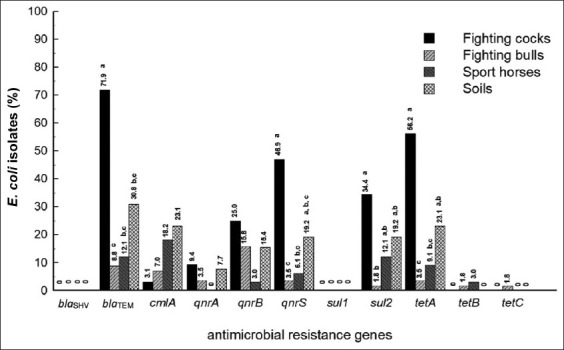
The frequency of antimicrobial resistance genes found in *Escherichia coli* isolated from fighting cocks (n = 32), fighting bulls (n = 57), sport horses (n = 33), and soils (n = 26). Different superscripts in each column mean statistically significant difference (p < 0.05).

The AMR genes and the significant difference (p < 0.05) among fighting cocks, fighting bulls, sport horses, and soils are indicated in Figure-2. For the eight resistance genes identified, the fighting cock isolates had the highest positive proportion, except for *cmlA, tetB*, and *tetC* that they had the highest positive proportion in soils, sport horses and fighting bulls, respectively. For *bla*TEM, *qnrS* and *tetA*, the positive rate in the fighting cock isolates was significantly higher than that found in the two other animal sources. The only resistance gene identified in fighting cocks at a level significantly above the soil isolates was *bla*TEM. No isolates yielded *bla*SHV or *sul1*, while only few isolates were positive for *tetB* and *tetC*. Regarding the resistance genes found in 20% or more of the *E. coli* isolates, those collected from fighting cocks harbored *bla*TEM (71.9%), *qnrB* (25%), *qnrS* (46.9%), and *tetA* (56.25%), whereas those obtained from soils carried *bla*TEM (30.8%) and *tetA* (23.1%).

## Discussion

*Escherichia coli* is a natural component of the intestinal flora of humans and animals [41]. *Escherichia coli* is excreted in feces and spreads quickly through food, water, and soil. While most *E. coli* strains are non-pathogenic, certain strains are primary pathogens capable of causing infections in people and animals, with enterohemorrhagic *E. coli* being an example of a form of *E. coli* that is a significant pathogen of humans and for which cattle constitute a major reservoir [42]. Antimicrobial resistance is a significant issue in human and animal medicine. Antimicrobial use at the farm level increases the likelihood of AMR development in commensal and pathogenic intestinal bacteria [2]. Our study was conducted to investigate *E. coli* isolates found in sports animals (fighting cocks, fighting bulls, and sport horses) and soils from their environment in terms of virulence genes, AMR patterns and genes, and genetic diversity.

Based on the phylogenetic results, most *E. coli* isolates from fighting cocks, fighting bulls, sport horses, and soils belong to Group B1 (40.6%, 59.7%, 72.7%, and 42.3%, respectively). Clermont *et al*. [32] showed that most extra-intestinal *E. coli* strains belong to Groups B2 and D, meaning that the *E. coli* isolates from sports animals in this investigation did not fall into the category of virulent extra-intestinal *E. coli*.

The five virulence genes used in this work were obtained from the previously identified virulent genes in avian pathogenic *E. coli* (APEC) and are associated with adhesion, iron acquisition, and serum resistance [31]. The highest prevalence of virulence genes was *iroN* (28.1%) and *hlyF* (25.0%) in *E. coli* isolates from fighting cocks. Overall, we found that this study’s prevalence of virulence genes in *E. coli* isolates was lower than that of pathogenic *E. coli* [43]. This shows that the *E. coli* isolates in this study were commensal *E. coli*, supporting the results of the phylogenetic grouping. In future work, it would be appropriate to use alternative sets of virulence genes that are known to be more relevant for the non-avian hosts, that is, the bovine and equine isolates.

Before starting this investigation, we hypothesized that antimicrobial drugs would be widely used to treat sports animals due to their high risk of injury and disease, causing the widespread use of antimicrobials in their treatment and a high prevalence of AMR. In fact, the AMR profile of *E. coli* isolates from sports animals and their environment was generally lower in this investigation than in earlier reports from other animals in Thailand, such as broilers, dairy cows, dogs, and cats [44–47]. The number of *E. coli* isolates resistant to AM, CF, and TE was significantly higher in the fighting cocks than in the others, suggesting that there may be some exposure to these antimicrobial agents. Interestingly, the resistance of beta-lactams and TE is similar to that of a previous study on *E. coli* isolates in broilers and native chickens [46], although this study had a lower occurrence. Antimicrobial resistance was less prevalent in *E. coli* isolates from fighting bulls, which may be attributed to the owner’s preference for natural medicine over contemporary medication (personal communication with owners).

Regarding samples from horses, there appears to be no previous report of the AMR pattern in equine *E. coli* isolates in Thailand. Antimicrobial resistance occurrence in *E. coli* isolates in Thai horses in this study seems lower than that reported in Turkey [48] and South Korea [49], but was similar to that reported in Japan [26]. The highest level of resistance in the equine *E. coli* isolates was against AM (15.2%), CF (12.1%), TE (12.1%), and SXT (9.1%). Beta-lactam antibiotics are among most used antimicrobial drugs in horses worldwide [50].

The only ESBL-positive isolate was found in the soil associated with fighting cocks. While only a single isolate was found, and none was found in the associated fighting cocks, it is possible that the birds were the source of the isolate through fecal soil contamination. Salah *et al*. [51] found ESBL-positive *E. coli* isolates in the environment.

In this study, 2 beta-lactamase genes, *bla*TEM, and *bla*SHV, were identified. *bla*TEM was the most prevalent in *E. coli* isolates from fighting cocks (71.9%), which agrees with recent studies on *E. coli* isolates from commercial broilers and native chickens [46, 52]. The detection rate of *bla*TEM in *E. coli* isolates from fighting bulls, sport horses, and soils were much lower at 8.8%, 12.1%, and 30.8%, respectively. According to Seo and Lee [53], the *bla*TEM gene encodes only narrow-spectrum beta-lactamases capable of inactivating penicillins and aminopenicillins. This is consistent with our findings, which showed marked resistance to AM and low resistance to cephalosporins. In addition, *bla*SHV was undetected in any isolate.

In this study, the *tetA* gene was the most predominant resistance gene found in *E. coli isolates* from fighting cocks (56.2%), fighting bulls (3.5%), sports horses (9.1%), and soils (23.1%). *tetB* was found only in isolates from fighting bulls (1.8%), and sports horses (3.0%), whereas *tetC* was found only in isolates from fighting bulls (1.8%). The *tetA* gene encodes efflux mechanisms and is the primary determinant of TE resistance in *E. coli* [33]. While *sul1* and *sul2* [54] deliver sulfonamide resistance, only *sul2* was found in isolates from fighting cocks (34.4%), fighting bulls (1.8%), sport horses (12.1%), and soils (19.2%). In contrast, Kim *et al*. [55] reported that most *E. coli* isolates contain the *sul2* gene.

While all *E. coli* isolates in this study were sensitive to ENR and MRB, both fluoroquinolones, the plasmid-mediated quinolone resistance genes (*qnrA*, *qnrB*, and *qnrS*) were identified from all groups, except that *qnrA* was absent in the equine *E. coli* isolates. These findings may be explained by the fact that *qnr* genes provide moderate resistance to fluoroquinolones in *Enterobacteriaceae* [51].

## Conclusion

This study has established that *E. coli* isolates from sporting animals and their environments can exhibit AMR. While the fighting cock isolates did not fit the APEC definition, these commensal *E. coli* containing AMR genes may play a significant role in transferring these genes to other bacteria and causing a zoonotic transmission due to humans close association with these sports animals. Monitoring of AMR in additional species of sports animals should be conducted in the future to assist in managing the emergence of AMR.

## Authors’ Contributions

TW and TT: Conceptualized and designed the study. TW, RN, NS, CS, YP, PF, and TT: Sample collection, materials, and analytic tools. TT and RN: Laboratory works. PS and TT: Statistical analysis and interpretation. TW, PJB, and TT: Wrote and revised the manuscript. All authors have read and approved the final manuscript.
